# Supplementary material for: Inhibition by Nitrogen Addition of Moss-Mediated CH4 Uptake and CO2 Emission Under a Well-Drained Temperate Forest, Northeastern China
Source: Plants (Basel). 2026 Jan 5;15(1):166. doi: 10.3390/plants15010166 (PMC12787504; doi:10.3390/plants15010166)
Supplement: Supplementary file 1 [file plants-15-00166-s001.zip › plants-4057745-supplementary.pdf]

Supplementary Material

Inhibition by nitrogen addition of moss-mediated CH_4 uptake and CO_2 emission under a well-drained temperate forest, northeastern China

Xingkai Xu[1,2*], Jin Yue[1], Weiguo Cheng[3], Yuhua Kong[4], Shuirong Tang[5], Dmitriy Khoroshaev[6], and Vladimir Shanin[6]

1 State Key Laboratory of Atmospheric Environment and Extreme Meteorology, Institute of Atmospheric Physics, Chinese Academy of Sciences, Beijing 100029, China yuejin@mail.iap.ac.cn (J.Y.)
2 Department of Atmospheric Chemistry and Environmental Science, College of Earth and Planetary Sciences, University of Chinese Academy of Sciences, Beijing 100049, China
3 Faculty of Agriculture, Yamagata University, Tsuruoka 997-8555, Japan; cheng@tds1.tr.yamagata-u.ac.jp (W.C.)
4 College of Forestry, Henan Agricultural University, Zhengzhou 450046, China; y.kong@henau.edu.cn (Y.K.)
5 School of Breeding and Multiplication (Sanya Institute of Breeding and Multiplication), Hainan University, Sanya 572025, China; tangshuirong@163.com (S.T.)
6 Institute of Physicochemical and Biological Problems in Soil Science of the Russian Academy of Sciences, Pushchino 142290, Russia; d.khoroshaev@pbcras.ru (D.K.), shaninvn@gmail.com (V.S.)

* Correspondence: xingkai_xu@mail.iap.ac.cn (X.X.).

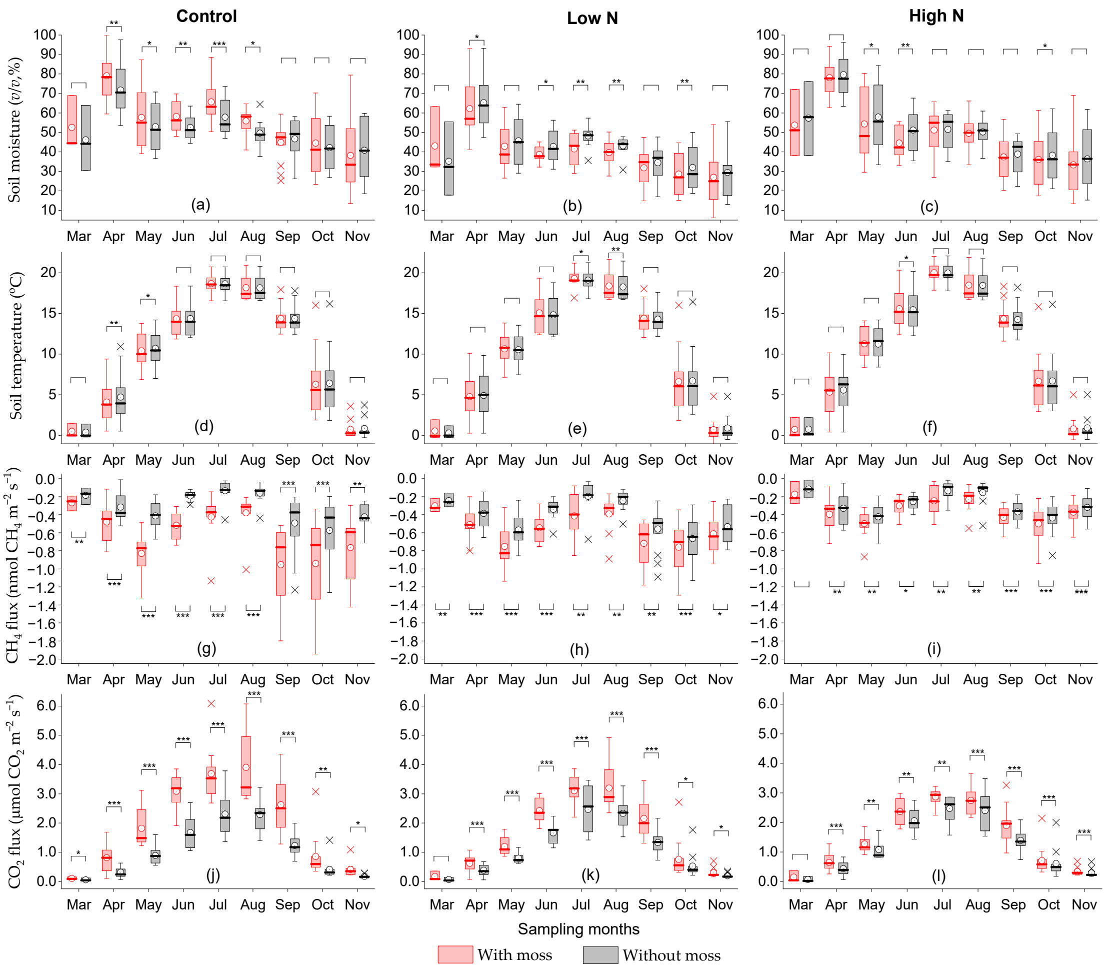

Figure S1: Box plots of monthly soil temperature (**a-c**) and moisture (**d-f**) at 7 cm depth, CH_4 (**g-i**) and CO_2 (**j-l**) fluxes from N-treated and non-treated plots with and without moss cover from March 2019 to November 2024. In winter (from December to early March) each year, no measurements were taken due to soil freezing and snow cover. Boxes show interquartile (IQR), and circles and horizontal lines in boxes show mean and median values, respectively. Lower and upper whiskers (x) represent 75 percentiles plus 1.5 IQR and 25 percentiles minus 1.5 IQR, respectively. *, $p < 0.05$; **, $p < 0.01$; ***, $p < 0.001$.

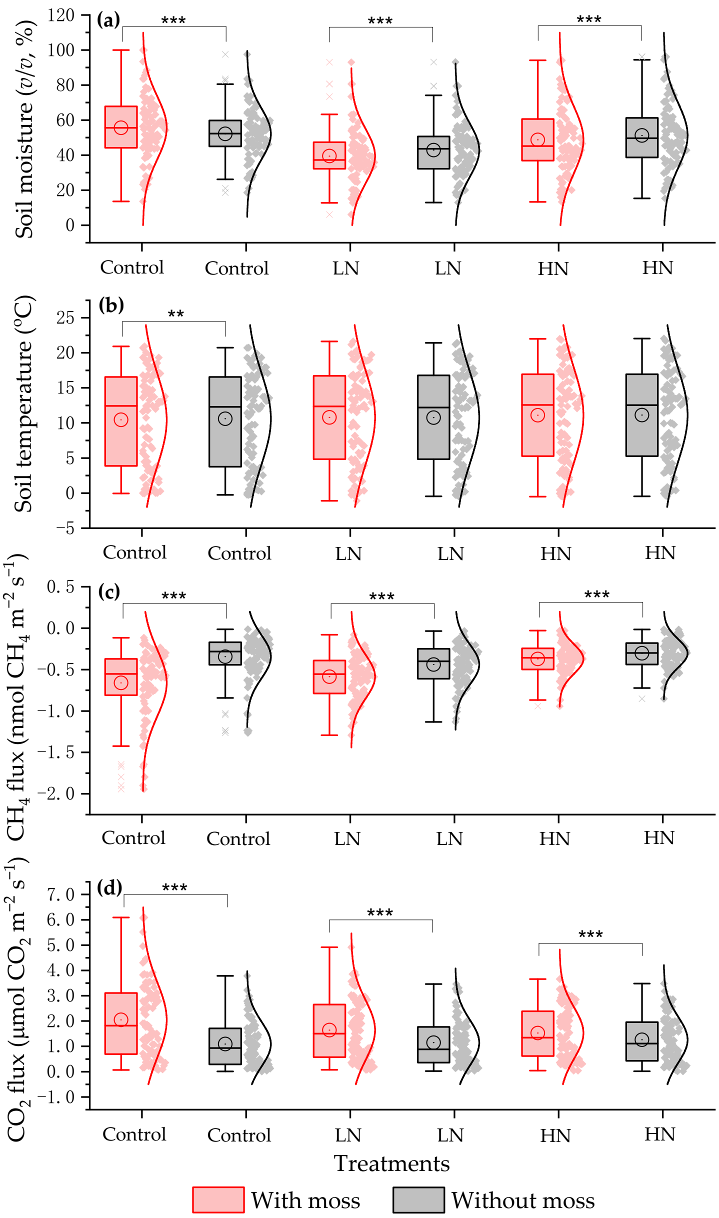

Figure S2: Box normal plots of daily soil moisture (**a**) and temperature (**b**) at 7 cm depth, daily CH_4 (**c**) and daily CO_2 (**d**) fluxes from N-treated and non-treated plots with and without moss cover from March 2019 to November 2024. Control, no N addition; LN, low N level; HN, high N level. **, $p < 0.01$; ***, $p < 0.001$. For the descriptions of the box plots, see the caption of Figure S1.

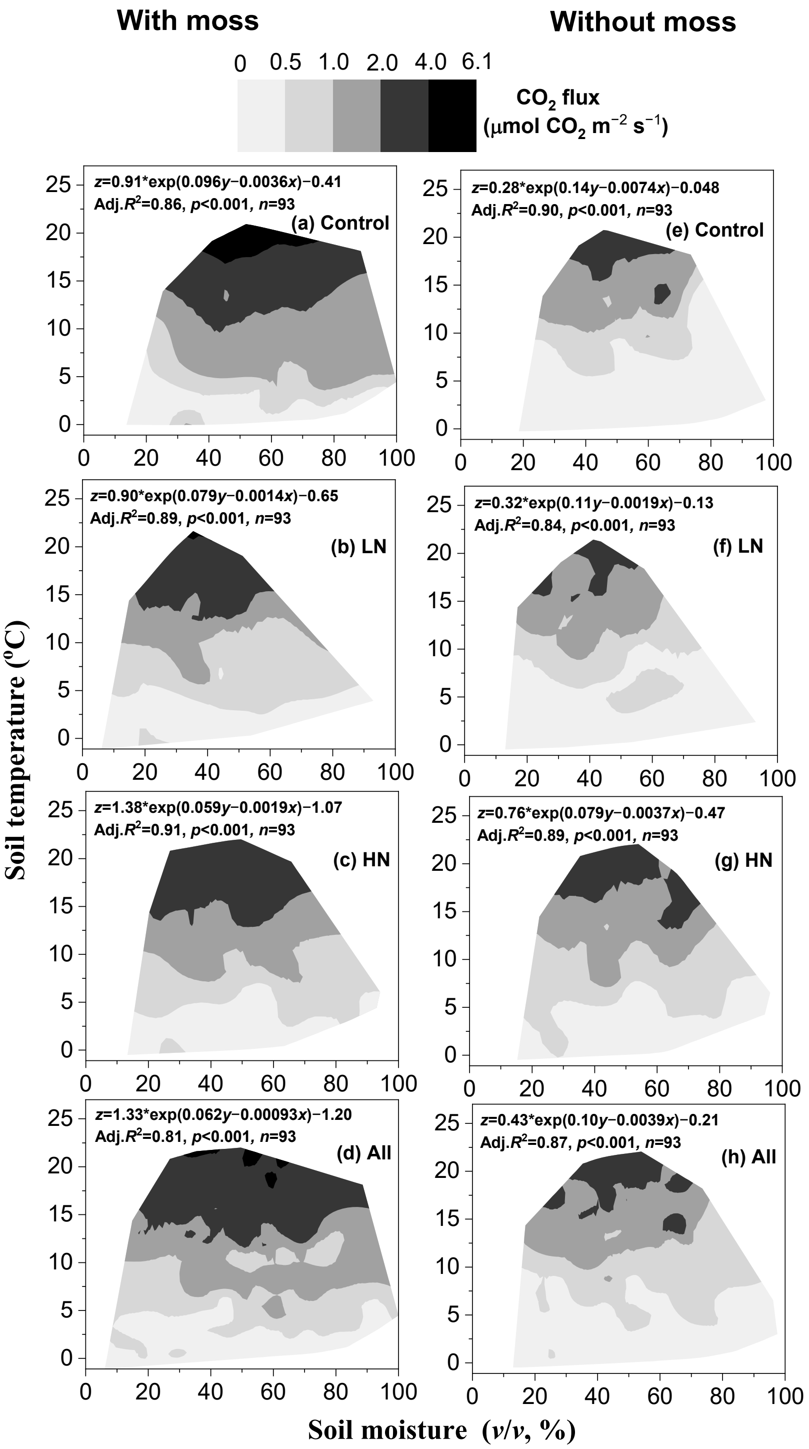

Figure S3: Combined effects of soil moisture and temperature at 7 cm depth in the N-treated and non-treated plots with and without moss cover on the daily CO_2 fluxes March 2019 to November 2024. The combined effects of soil moisture (x) and soil temperature (y) on daily CO_2 fluxes (z) were fitted with multivariate regression curves. Control, no N addition; LN, low N level; HN, high N level; All; all treatments. Legends with different gray scales show the different ranges of daily CO_2 fluxes.

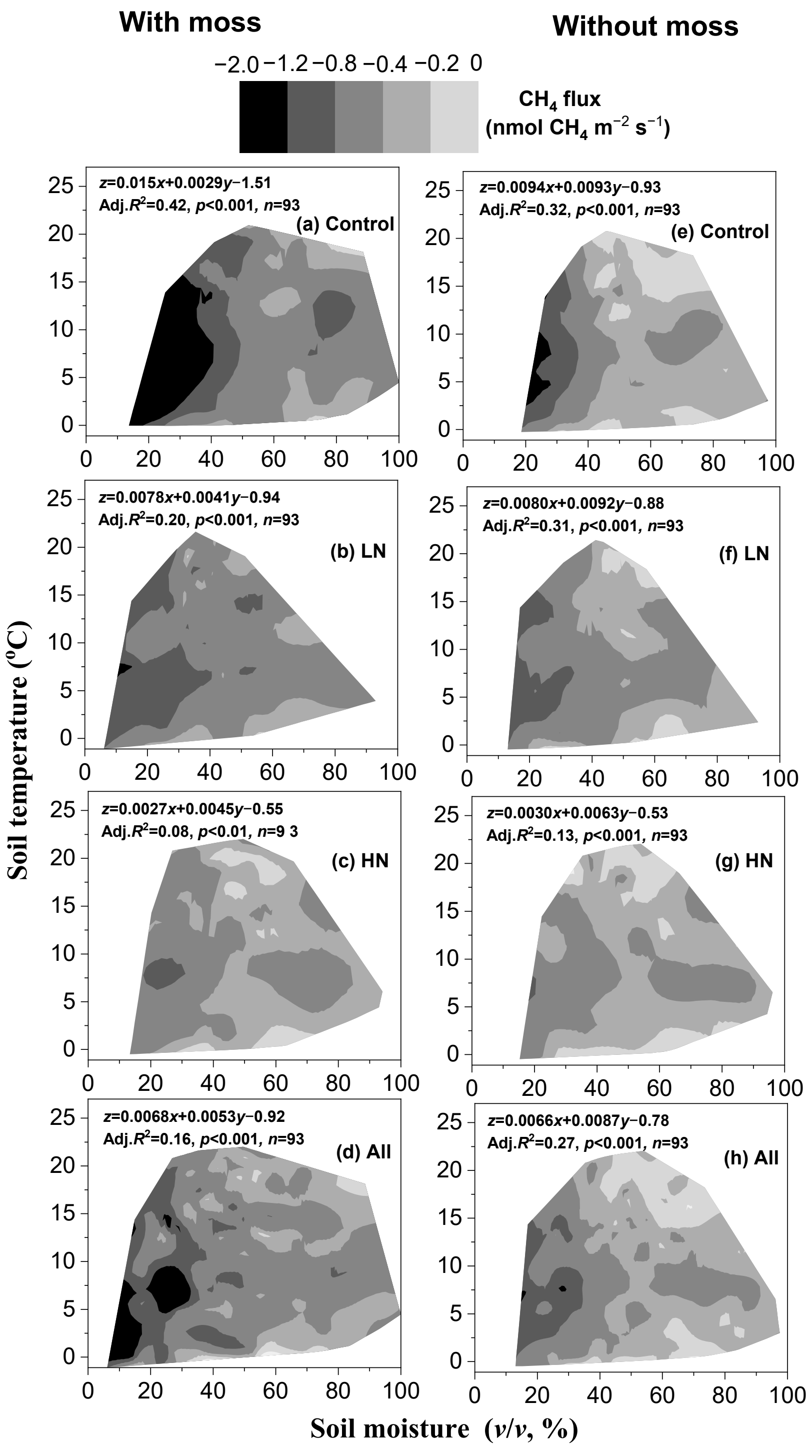

Figure S4: Combined effects of soil moisture and temperature at 7 cm depth in the N-treated and non-treated plots with and without moss cover on the daily CH_4 fluxes from March 2019 to November 2024. The combined effects of soil moisture (x) and soil temperature (y) on daily CH_4 fluxes (z) were fitted with multivariate regression curves. Control, no N addition; LN, low N level; HN, high N level; All; all treatments. Legends with different gray scales show the different ranges of daily CH_4 fluxes.

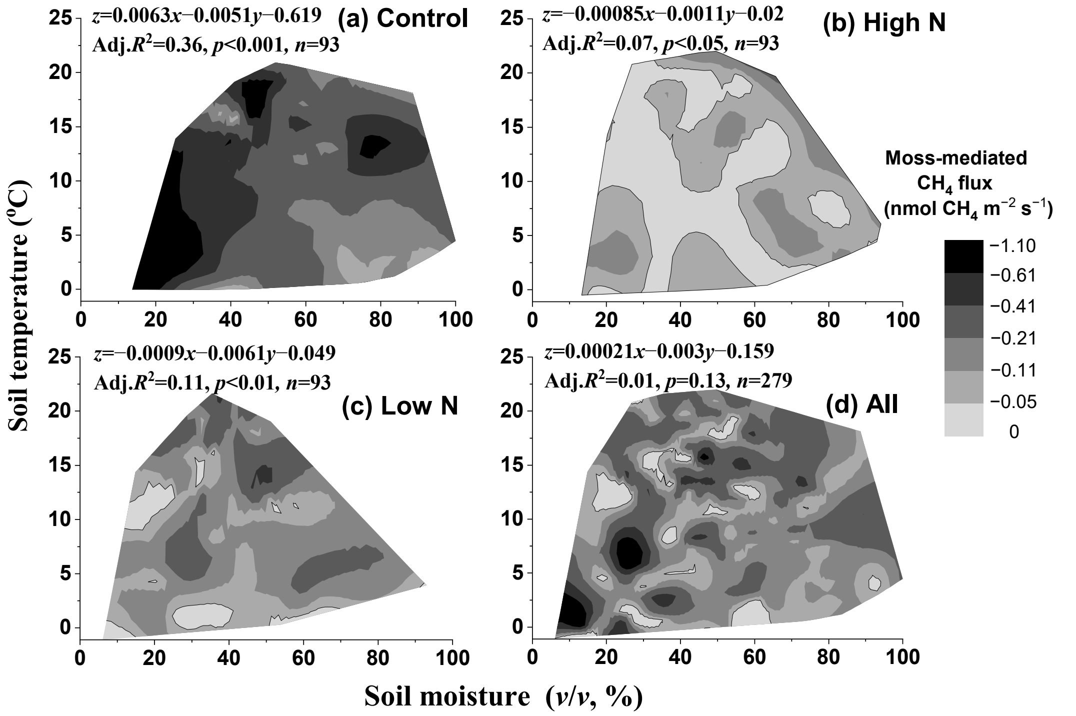

Figure S5: Combined effects of soil moisture and temperature at 7 cm depth in the N-treated and non-treated plots with moss cover on the daily moss-mediated CH_4 fluxes March 2019 to November 2024. The combined effects of soil moisture (*x*) and soil temperature (*y*) on the daily moss-mediated CH_4 fluxes (*z*) were fitted with multivariate regression curves. Legends with different gray scales show the different ranges of daily moss-mediated CH_4 fluxes.

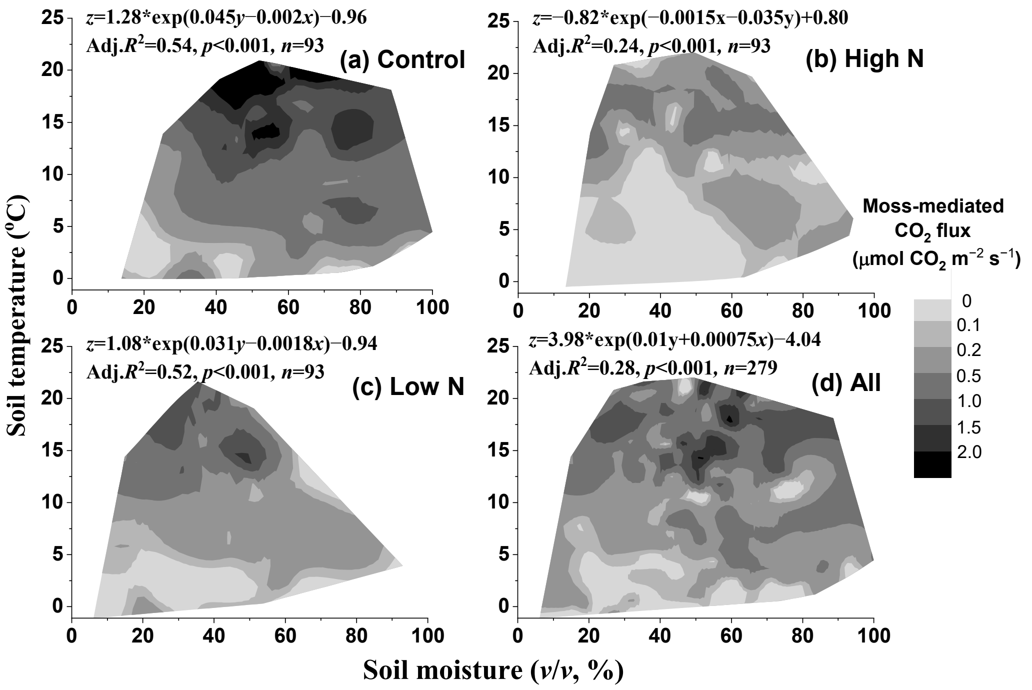

Figure S6: Combined effects of soil moisture and temperature at 7 cm depth in the N-treated and non-treated plots with moss cover on the daily moss-mediated CO_2 fluxes March 2019 to November 2024. The combined effects of soil moisture (x) and soil temperature (y) on the daily moss-mediated CO_2 fluxes (z) were fitted with multivariate regression curves. Legends with different gray scales show the different ranges of daily moss-mediated CO_2 fluxes.

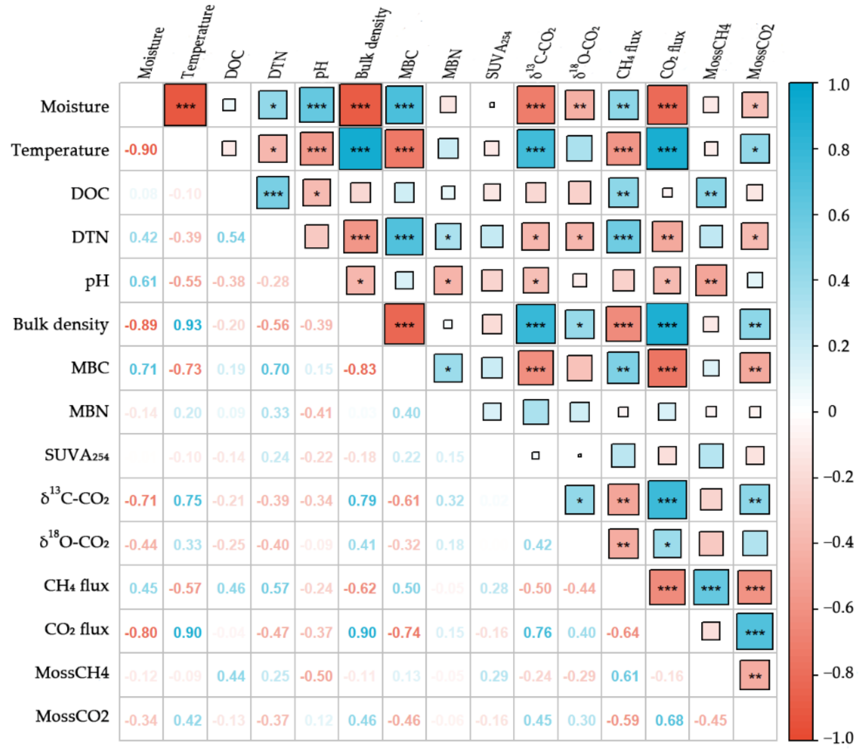

Figure S7: Pearson correlation coefficients between moss-mediated CO_2 and CH_4 fluxes, CO_2 and CH_4 fluxes, and soil environmental variables in the N-treated and non-treated plots with mosses. MossCO2, moss-mediated CO_2 flux; mossCH4, moss-mediated CH_4 flux; DOC, dissolved organic C; DTN, dissolved total N; MBC, microbial biomass C; MBN, microbial biomass N; $SUVA_{254}$, the special UV values at 254 nm of soil K_2SO_4-extracts. Both $\delta^{13}C$-CO_2 and $\delta^{18}O$-CO_2 represent $\delta^{13}C$ and $\delta^{18}O$ values of carbon dioxide released from N-treated and non-treated plots with mosses, respectively. *, $p < 0.05$; **, $p < 0.01$; ***, $p < 0.001$.

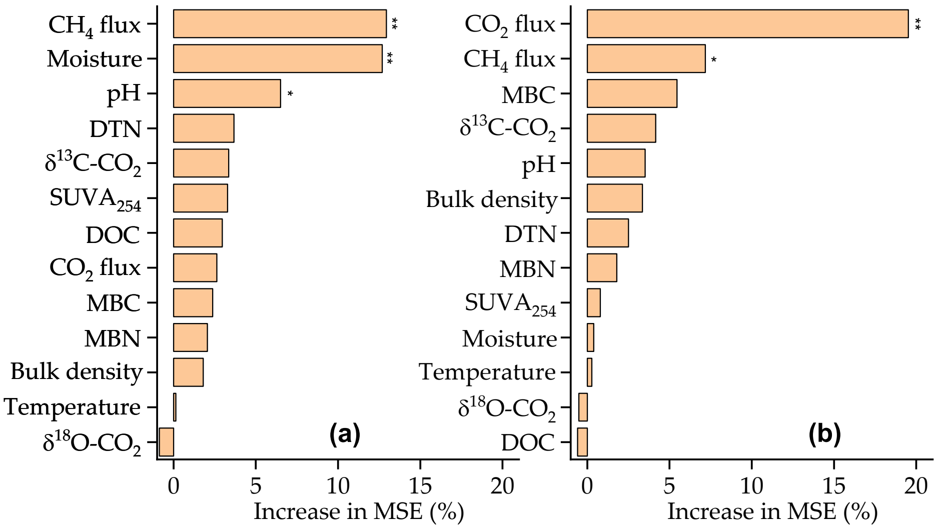

Figure S8: Random forest analysis explaining the effects of environmental variables on the moss-mediated CH_4 (R^2 = 0.73, (**a**)) and CO_2 (R^2 = 0.62, (**b**)) fluxes. MSE, mean squared error; CH_4 and CO_2 fluxes represent CH_4 and CO_2 fluxes from all N-treated and non-treated experimental plots with mosses; other abbreviations are shown in the caption of Figure S7. *, $p < 0.05$; **, $p < 0.01$.

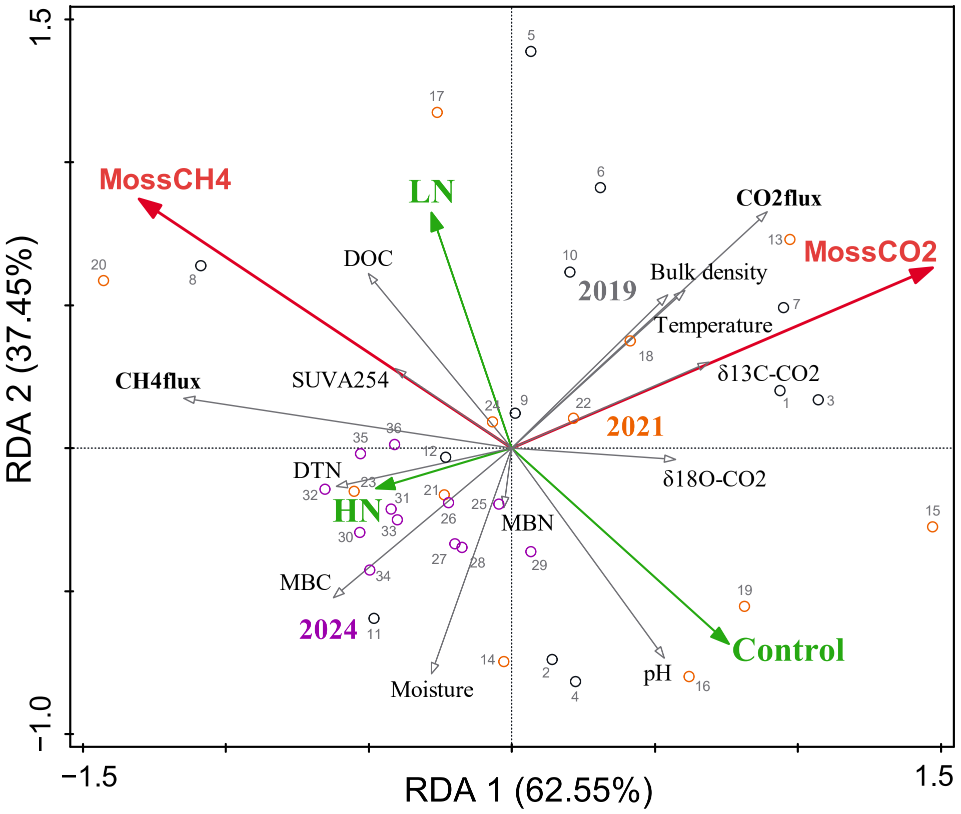

Figure S9: Biplot of redundancy analysis (RDA) for the relationships among CO_2 fluxes, $\delta^{13}C$-CO_2 and $\delta^{18}O$-CO_2 values, CH_4 flux, soil properties in the experimental plots with mosses, and moss-mediated CO_2 and CH_4 fluxes. Control, no N addition; LN, low N level; HN, high N level; other abbreviations are shown in the captions of Figure S7. Circles in gray, green, and purple represent soil samples collected in 2019, 2021, and 2024, respectively.
